# Designing Novel Endodontic Implants and Evaluation of the Stress Distribution in Maxillary Anteriors Using Finite Element Analysis

**DOI:** 10.7759/cureus.62562

**Published:** 2024-06-17

**Authors:** Shruthi Rajagopal, Sonali Sharma

**Affiliations:** 1 Department of Conservative Dentistry and Endodontics, Saveetha Dental College and Hospitals, Saveetha Institute of Medical and Technical Sciences, Saveetha University, Chennai, IND

**Keywords:** anterior fractures, bioceramics, finite element analysis (fea), titanium, maxillary anterior teeth, endodontic implant

## Abstract

Introduction

Endodontic implants, or didontic implants, offer a promising solution for stabilizing compromised teeth with a guarded prognosis and prolonging their clinical survival rate. Despite their potential benefits, they retired out of practice due to failures that arose from the lack of a biocompatible seal and engaging in dentin. Novel designs, based on evidence-based research with the help of bioceramics, present an opportunity to overcome these challenges and hence, enhance the clinical efficacy of endodontic implants. Thus the aim of this study is to design novel endodontic implants and evaluate their stress distribution in maxillary incisors using finite element analysis (FEA).

Materials and methodology

FEA is a biomechanical study to assess the stress distribution and extent of displacement to assess the clinical efficacy of novel endodontic implants in maxillary anterior teeth. Three 3D models (Model 1, Model 2, and Model 3) are designed to be meshed, and material elastic properties of the tooth and periapical tissues are applied. Boundary conditions were established, and a constant axial load value of 600 N was applied at a 45° angle. The FEA analysis was done under the loading conditions to assess the stress patterns for the three 3D models in comparison to the intact tooth on the ANSYS software (ANSYS Inc, Pennsylvania).

Results

FEA simulations revealed the distribution of stress within the tooth structure under functional occlusal forces, as Von Misses stresses were analyzed to assess the likelihood of material yielding and failure, which was comparable to that of an intact tooth. The maximum stress of deformation was as follows: intact: 1.7589e-5 MAX; Model 1: 3.3804e-6 MAX; Model 2: 2.638e-5 MAX; and Model 3: 2.1986e-5 MAX. The area of stress concentrations did not occur at the interface of the coronal or apical seal, which prevented catastrophic failures.

Conclusion

By leveraging advanced design principles and materials, these implants offer a promising alternative to traditional approaches, particularly in trauma cases with a poor prognosis for the survival of the teeth leading to loss of tooth. Further clinical studies are warranted to validate the efficacy and long-term success of these novel endodontic implants in diverse patient populations.

## Introduction

The concept of minimal intervention in dentistry is the current outlook for practitioners across the globe. Keeping that in mind, the need for the preservation of teeth especially in the anterior region regardless of the prognosis is critical. Often in certain clinical scenarios, teeth having poor prognosis are referred for extraction followed by prosthetic rehabilitation. The primary reason is due to the lack of an alternative option.

Didontic implants imply "through the tooth" in Greek. They are extensions composed of metals that may extend past the tooth's apex and into the underlying healthy bone, also known as endodontic endosseous implants [1]. A time-tested method for stabilizing periodontally weak teeth has been provided by the endosseous implants' capacity to lengthen roots, change the ratio of root to crown measurement, support roots for overdentures, immobilize shattered or resorbed roots, and stabilize periodontally impaired teeth [2]. Though first advocated by Orlay among the others, Frank was credited for setting a protocol in place, with standardized recommendations for the choice of endodontic implant size depending on the clinical situation [3]. These initial designs were composed of sapphire, chrome-cobalt, and vitalium and were first described in 1960 [4]. Wein et al. were the first to introduce designs like threaded and non-threaded implants, which provided various amounts of retention and inadvertently led to dentin crazing. In 1963, the Strock technique was developed that involved making these posts with titallium or vitallium, extending 5 mm beyond the apex. Endodontic implants, as treatment modalities, are pivotal in retaining teeth with a hopeless prognosis that could occur due to trauma or unfavorable periodontal conditions. Teeth with endodontic implants are known to have prolonged rates of survival. After a five-year follow-up, the prognosis for endodontic implants was reported to be as high as 91% [5].

Wein and Frank conducted a 10-year follow-up of their cases, and while there were several failures, there were also some clinical successes ranging up to even 15 years of follow-up. Keeping in mind, that the prognosis to begin with involves the inevitable loss of such teeth, any amount of extended stabilization delaying this can be considered a success from the patient's point of view. The more common causes of such failures were a consequence of a variety of factors [6]. Clinical factors such as occlusion, amount of tooth structure, quality of dentin, and surrounding bone influenced case selection for this treatment modality, which were often not considered. The materials used for the development of endodontic implants were not the most bio-inert and biocompatible. Moreover, the designs did not take into consideration the stress distribution patterns prior to the preparation of the teeth, and accepting such restorations led to either over-preparation or under-preparation. The early endodontic implants were cemented with zinc oxide eugenol or eugenol-free cement, which were non biocompatible with the periapical tissues. Hence with the rise and strides made in the science of osseointegrated implants in dentistry, the use of endodontic stabilizers became discouraged [7,8].

With the advent of biocompatible materials like titanium for implant manufacture and the development of bioceramic calcium silicate cement, the clinical efficacy of endodontic implants holds great promise [9]. There is now a wide range of surface possibilities for endodontic implants that may enhance their application and long-term clinical success. Improvement in biocompatibility combined with the ability to preserve periodontal attachment makes them a good alternative, as a treatment modality for preserving natural dentition.

Finite element analysis (FEA) is a widely used method for studying dental biomechanics by dividing the geometry into small elements with known mechanical properties. Based on the concept of "moving from part to whole," this approach breaks the topic under investigation into digestible sections. The three main steps of the FEA are preprocessing, which prepares the modeling data, processing of the assembly that solves the equations, and postprocessing, which visualizes the findings after analysis [10]. The rheological and physical characteristics of biological tissues and biomaterials are used to construct a model. To be more precise, figures pertaining to the modulus of elasticity, stress, and Poisson's ratio of the material are required for the final construction. The output of FEA is expressed using various stresses, such as tensile, compressive, shear, or a combination known as Von Misses stresses. When a complex loading condition is applied to an isotropic and ductile material, the Von Misses stress is typically employed to determine if the material will yield. Principle stress, on the other hand, is the highest and lowest normal stress on a principle plane when there is no shear stress applied to a body. Though it is frequently inaccurate for ductile materials, the maximum primary stress theory is more reliable in predicting failure, particularly in brittle materials. Thus, von Misses stresses were taken into account in this investigation [11,12]. Based on the condition that all the parts are connected to one another by the nodes, it is possible to estimate the stresses, the variables that develop, and the overall deformation produced in each node. Because teeth are asymmetrical, reliable research requires the 3D reproduction of the alveolar bone, periodontal ligament, and numerous other essential components to simulate the oral cavity [13].

Thus in order to revive the concept of endodontic implants, as an alternative to preserving teeth with poor prognosis, novel designs need to be developed keeping in mind the widely used successful strategies of osseointegrated dental implants. These designs need to incorporate various modifications that can be cemented with calcium silicate cement with ease while promoting stress distribution to the surrounding bone minimizing catastrophic fractures of the teeth. Hence, the aim of this study was to design novel endodontic implants and to evaluate the stress distribution under functional loading in maxillary anterior teeth by using FEA.

## Materials and methods

This pre-clinical software-based study was conducted at Saveetha Dental College, Chennai. As mentioned the objective of this study is to design endodontic implants keeping the current successful systems of osseointegrated implants in mind. It was proposed that after the healing phase of an endodontic implant, there is the development of a periodontal ligament-like structure around it at the level of the apex of the tooth [14].

Requirements

Keeping this in mind as the end goal for functional healing, the design of the endodontic implant models requires the following: threads that would stabilize the endodontic implant, but not concentrate stress and cause crazing in the dentin; sluiceway for the cement to flow and express through the coronal aspect; matched major diameter at the level of the apex in order to prevent root fracture; smooth surface beyond the apex in order to connect with the osteostimulatory collagen fibers for osteopreservation; and the head of the implant to match the coronal prepared space and taper with the canal.

Designs of endodontic implants

The representation of all the dimensions of the novel designs is mentioned for Model 1, Model 2, and Model 3 in Table 1.

**Table 1:** Table representing the dimensions applied for designing the various models of endodontic implants

Dimensions (mm)	Model 1 (mm)	Model 2 (mm)	Model 3 (mm)
Length of the head	4	3	0.5
Length of the shaft	6	3	0.5
Length of screws	4	11	12
Length of the apical extension	4	4	4

Model 1 

**Figure 1:**
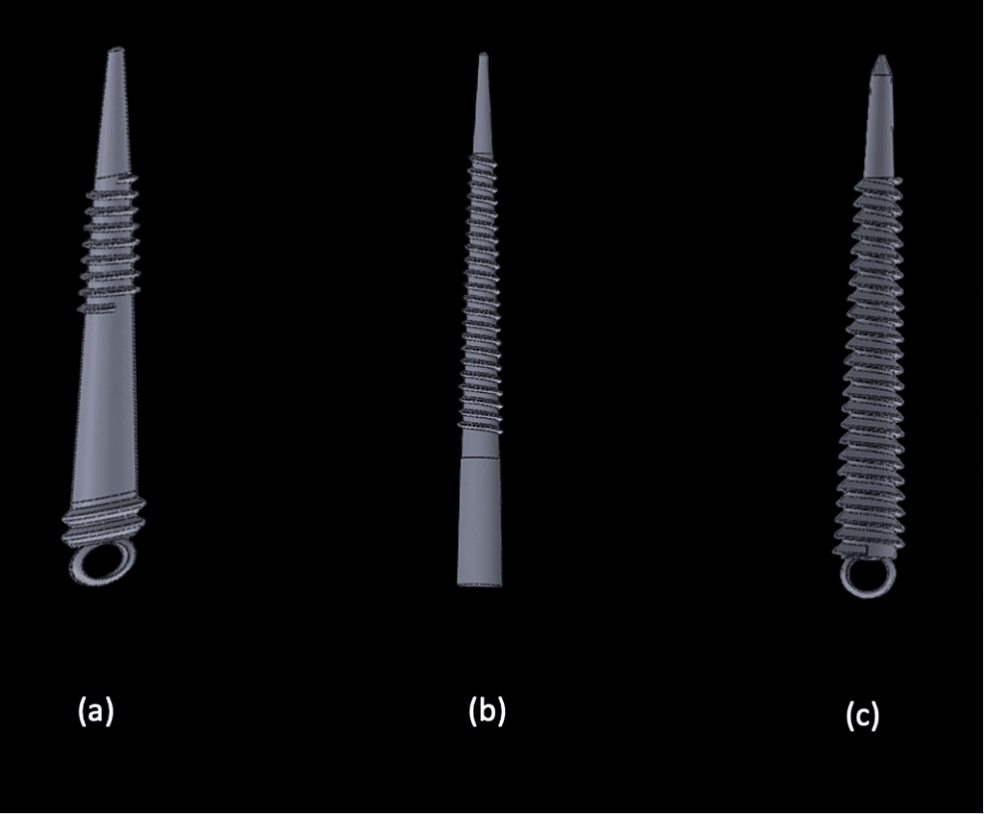
(a) represents Model 1 with spiral thread design, (b) represents Model 2 with buttress thread design, and (c) represents Model 3 with reverse buttress thread design. The 3D figures were rendered in SpaceClaim (SpaceClaim Corp., Massachusetts)

The pictographic design had been designed first with the dimensions as represented in Table 1. The thread was designed as a spiral thread and the head was circular in order to screw it in the tooth structure. The threads are placed at the junction of the middle and apical third of the implant and kept smooth otherwise in order to decrease stress in the dentin walls. The digital model for the same is represented in Figure 1.

*Model 2* 

The pictographic design had been designed first with the dimensions as represented in Table 1. The thread was designed as a buttress thread and the head was made parallel in order to screw it in with a hex into the tooth structure. Except for the apical and coronal 4 mm, threads were placed throughout for increased retention. The digital model for the same is represented in Figure 1. 

Model 3 

The pictographic design had been designed first with the dimensions as represented in Table 1. The thread was designed as a reverse buttress thread and the head was made circular in order to screw it into the tooth structure. Except for the apical 4 mm, the endodontic implant had threads throughout. The reverse buttress thread would not engage the dentin walls to a great extent and hence, in order to increase the surface area of adaptation the design was finalized. The digital model for the same is represented in Figure 1. 

**Figure 2:**
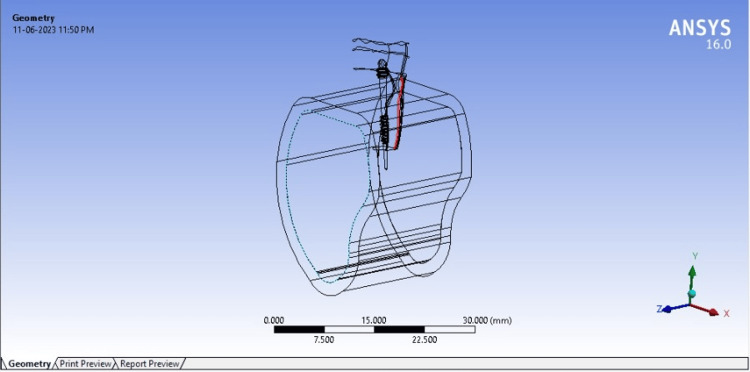
Figure representing a schematic model (ANSYS software) of maxillary central incisor, alveolar bone, and endodontic implant placed within the apical extension. The head is placed at the level of the cingulum palatally

After the designing and exporting of the models, a computer-simulated tooth model was composed of separate components connected at nodes, which were formed from the material properties. Meshing was done after the model had been constructed and boundary conditions were established to ensure that the body under assessment was confined when stress was applied. The alveolar bone along with the maxillary central incisor were the first parts of the model to be created. This is represented in Figure 2.

These models represent conditions similar to the oral cavity and are simulated and built using evidence-based scientific data to estimate the mechanical properties of materials, such as Poisson ratio and Young's modulus. It was assumed that all materials were linearly elastic, homogenous, and isotropic. The models were meshed, boundary conditions were created, and the load was measured on the palatal region 2 mm below the incisal edge; at a 45° angle, a constant axial load value of 600 N was applied after the application of a boundary condition. The next step was to do 3D FEA using ANSYS software (ANSYS Inc, Pennsylvania) [15].

The workflow for obtaining the FEA analysis of the final models is as follows: preparation of maxillary bone model; maxillary central incisor model; assembly; creation of periodontal ligament; separation of cortical and cancellous bone; creation of enamel, dentine, and pulpal space; creation of endodontic implant models; creation of endodontic implant beyond root apex; application of material properties; meshing and application of boundary settings; and analysis of the given occlusal loads in intact and clinical scenarios. 

These models represent conditions that are emulated from the oral cavity, and hence, the simulations are built using evidence-based scientific data to estimate the mechanical properties of materials, such as Poisson ratio and Elastic modulus as represented in Table 2.

**Table 2:** The elastic properties of the dental and periapical tissues along with the implant material are represented in the table used for FEA analysis FEA: finite element analysis

Materials	Elastic modulus (Gpa)	Poisson’s ratio
Enamel	93	0.3
Dentine	18.6	0.31
Pulp	0.002	0.45
PDL	0.0689	0.45
Alveolar bone	11.5	0.3
Cortical bone	13.7	0.3
Titanium alloy	80	0.31

The results obtained are then inspected and studied to understand the extent and pattern of stress distribution in Model 1, Model 2, and Model 3 in comparison to that of an intact tooth.

## Results

**Figure 3:**
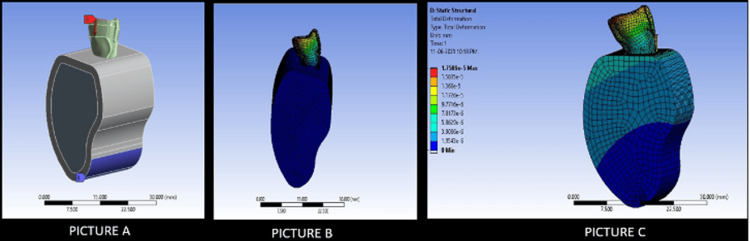
The picture depicts the novel 3D model of Model 1 (ANSYS software). Picture A depicts the stress applied on the incisal edge to study stress generation (70 N). Picture B depicts the total stress generated on the tooth structure after the placement of the endodontic implant of Model 1. Picture C depicts the stress generated on an intact model

The FEA was done for all three models, which showed that the maximal stress concentration is on the incisal edge. This is a good indication, as the weakest point is not situated at the junction between the endodontic implant and the coronal/apical region of the tooth, potentially preventing catastrophic failures during clinical situations. Figure 3 depicts the stress distribution in Model 1 in comparison to that of an intact tooth.

**Figure 4:**
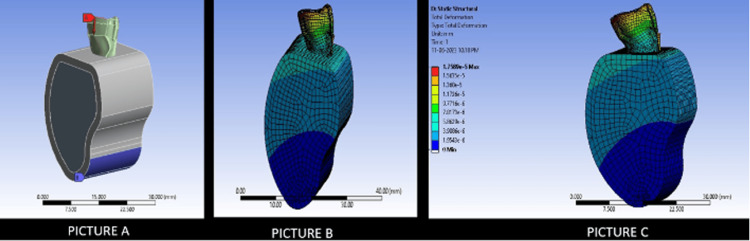
The picture depicts the novel 3D model of Model 2 (ANYSYS software). Picture A depicts the stress applied on the incisal edge to study stress generation (70 N). Picture B depicts the total stress generated on the tooth structure after the placement of the endodontic implant of Model 2. Picture C depicts the stress generated on an intact model

Figure 4 depicts the stress distribution in Model 2 in comparison to an intact tooth.

**Figure 5:**
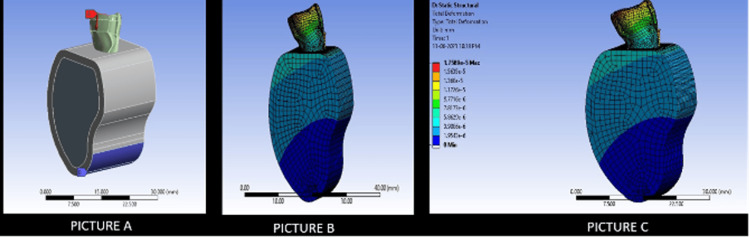
The picture depicts the novel 3D model of Model 3 (ANSYS Software). Picture A depicts the stress applied on the incisal edge to study stress generation (70 N). Picture B depicts the total stress generated on the tooth structure after the placement of the endodontic implant of Model 3. Picture C depicts the stress generated on an intact model

Figure 5 depicts the stress distribution in Model 3 in comparison to an intact tooth.

Table 3 depicts the stress distribution and maximum stress obtained with FEA analysis with ANSYS software after loading for all three models.

**Table 3:** The stress level of deformation obtained through ANSYS software on applying the functional loads for all three models

Model	Max. stress of deformation
Intact	1.7589e-5 MAX
Model 1	3.3804e-6 MAX
Model 2	2.638e-5 MAX
Model3	2.1986e-5 MAX

## Discussion

Preserving and extending the longevity of the natural dentition is the priority whenever possible even in clinical cases that produce guarded prognosis. According to Frank and Abrams, an appropriately positioned endodontic implant is accepted by the periapical tissue, where the titanium metal implant is encircled by a thin, healthy fibrous connective tissue "collar" that resembles a periodontal ligament-like tissue of natural teeth [16]. This hardened membrane-like structure is a mode of tissue integration that becomes a channel of stress distribution of the functional loads to the surrounding bone and is composed of osteostimulatory collagen fibers. This is called “osteopreservation” [17].

When it was introduced, the endodontic implants were made of materials that were not completely biocompatible with the periapical tissues. They caused tissue reactions that were not conducive to osteopreservation. This was one of the critical reasons for the failure of endodontic implants in clinical situations. The introduction of titanium along with variations like titanium alloys with vanadium and aluminum has opened the door for various grades of materials that are bio-inert and biocompatible. Hence, this study was conducted to design novel designs of endodontic implants in order to re-introduce them in contemporary clinical dentistry using titanium as the material of choice [18,19].

In order to formulate these designs, the points and reasons for the failures of the previous systems have to be identified. Hence, previously recorded case reports by multiple authors were studied, which is represented in Table 4.

**Table 4:** Cases reports representing the utilization of endodontic stabilizers in clinical scenarios along with details of the studies

Author	Treatment details	No. of cases reported	Remarks
Yadav RK et al. (2014) [20]	Used reamer and H-file as endodontic implants	2 cases, 6-month follow-up was done	Patients with reamer used as a stabilizer reported less mobility. In patient with H file used as a stabilizer reported reduced mobility and bone formation in the previous radiolucent area
Mittal et al. (2011) [21]	18 mm endodontic endosseous stabilizer	Follow-up at 1 week, 3 months, and 6 months	The results showed excellent bone formation around the endodontic stabilizer
Priyadarshini L (2000) [22]	No. 100 file threaded into the prepared channel	One patient reviewed after 1 week and followed every month and after 6 months of follow-up	Authors reported appreciable bone formation around the implant and a complete reduction in tooth mobility
Larsen et al. (1989) [23]	Vitallium endodontic implants	2 cases followed up for 5 years	Endodontic implants can successfully improve the crown-root ratio of central incisors compromised by trauma. Further authors reviewed recent developments in endodontic implant materials and designs and concluded that new materials should provide greater biocompatibility and retention

The proposed causes of failure of these previous implant designs were attributed mainly to lack of achievement of the apical seal, screwing-in of the implant within the tooth, along with crazing and crack propagation in the dentine, and finally poor retention in the bone beyond the root apex.

As seen with the results in the FEA, the stress patterns found in the tooth models were favorable and comparable to those seen in an intact tooth. The overall maximum stress of deformation was also comparable to that seen in an intact tooth. In non-threaded implants, the apical seal was highly dependent on the wedging of the implant, along with the cement used at the apical dentine interface. However, with an irregular apical opening and circular implant, sealing the interface would be most difficult if not impossible. Considering all these factors, Model 2 and Model 3 were modeled after the current best implant systems with meticulous designing of the apical end [24]. The FEA analysis noted that all models show maximal failure on the incisal edge, which shows that the pattern of failure will not be in the apical end and hence, preventing all kinds of catastrophic failures. Furthermore, Model 3 had the closest stress distribution pattern comparable to an intact tooth. This is because reverse buttress thread designs have been seen to distribute the stress in an even manner without concentrating the stresses at certain points.

Hence, the novel designs presented in this study provide favorable results that can be extrapolated to clinical settings after further future developments. This study has a few limitations. The main limitation is that only one model of each of the designs was compared to the intact tooth model. The conditions replicated represented all the physical aspects of the materials and tooth included in an ideal setting, but not an exact representation of the oral cavity. Hence, there should be more clinical studies comparing different tooth designs and different designs of endodontic implants to study their longevity.

## Conclusions

Endodontic implants, although having a mixed success rate in the past, are now a reliable choice for the treatment of maxillary anterior teeth, particularly in trauma cases with a poor crown-root ratio, root resorption, and apical resorption due to the development of biocompatible materials and cement. All three novel designs showed comparable stress patterns in comparison to an intact tooth. The most promise was shown by Model 3 with a reverse buttress thread design running throughout the length of the endodontic implant except for a smooth apical extension. Future studies are to be conducted to assess the feasibility of these designs in clinical conditions.
